# Elevated BNP caused by recombinant human interleukin-11 treatment in patients with chemotherapy-induced thrombocytopenia

**DOI:** 10.1007/s00520-019-04734-z

**Published:** 2019-03-15

**Authors:** Na-wei Liu, Xin Huang, Shuang Liu, Wen-jian Liu, Hua Wang, Wei-da Wang, Yue Lu

**Affiliations:** 1 Department of Hematologic Oncology, Sun Yat-sen University Cancer Center, Guangzhou, China; 2 Center State Key Laboratory of Oncology in South China, Guangzhou, China; 3 Collaborative Innovation Center for Cancer Medicine, 651 Dongfeng East Road, Guangzhou, 510060 People’s Republic of China; 4 Department of Hepatobiliary Oncology, Sun Yat-sen University Cancer Center, Guangzhou, China

**Keywords:** Thrombocytopenia, Chemotherapy, Recombinant human interleukin-11 (rhIL-11), Brain natriuretic peptide (BNP), Heart failure

## Abstract

Thrombocytopenia is a condition characterized by abnormally low levels of thrombocytes and often induced by chemotherapy. Recombinant human interleukin-11 (rhIL-11) is a cytokine that can stimulate thrombopoiesis and is commonly used to treat thrombocytopenia. We observed the side effects of rhIL-11 in 24 leukemia patients with chemotherapy-induced thrombocytopenia. To determine the cardiovascular effects of rhIL-11, we detected changes in the patients’ serum brain natriuretic peptide (BNP), blood pressure fluctuations, weight change, and whether edema or heart failure occurred in leukemia patients after chemotherapy. The results showed that BNP was significantly elevated after using rhIL-11 (*P* < 0. 05) but regressed after 2–4 days. Furthermore, nine patients had edema and experienced weight gain, and four experienced acute left heart failure. In addition, the average blood pressure was 119/75 mmHg (range 139/86 mmHg to 99/64 mmHg) before rhIL-11 administration and 127/79 mmHg (range 146/89 mmHg to 108/69 mmHg) after rhIL-11 use. In conclusion, although rhIL-11 is useful for treating chemotherapy-induced thrombocytopenia, it is important to monitor the patients’ clinical status and re-examine BNP levels frequently during the use of rhIL-11. Furthermore, senile patients should be given special attention. However, the appropriate timing to begin and discontinue rhIL-11 treatment needs further investigation.

## Introduction

Acute leukemia (AL) is a malignant clonal hematopoietic disorder that originates from genetic alterations in healthy hematopoietic stem cells. These affected cells multiply vigorously in the bone marrow and other hematopoietic tissues and can further infiltrate nonhematopoietic tissues and organs while suppressing normal hematopoietic processes. The clinical manifestations of AL include various degrees of anemia, bleeding, infection, hepatosplenomegaly, lymphadenectasis, and bone pain. AL is a highly malignant systemic disease, and the preferred treatment is systemic chemotherapy, which can cause severe bone marrow suppression. Platelet reduction is one of the main manifestations of bone marrow suppression in patients. Thrombocytopenia can result in mucocutaneous hemorrhage and may even cause bleeding in vital organs that is life-threatening to patients.

The human IL-11 gene is located on chromosome 19. The protein encoded by this gene is a multipurpose cytokine that was first detected in bone marrow-derived stromal cells in 1990. IL-11 is a member of the IL-6-type cytokine family, which is distinguished by their interaction with the common coreceptor gp130 and plays an important role during the hematopoiesis process. Furthermore, the IL-6-type cytokine family has a synergistic effect on a variety of cytokines, with one of the most notable effects being the stimulation of megakaryocyte maturation resulting in increased platelet production [1]. Recombinant human interleukin-11 (rhIL-11) is currently undergoing a series of clinical trials and is used as a preferred drug to treat chemotherapy-induced thrombocytopenia [2–4].

Brain natriuretic peptide (BNP) is a natural hormone secreted by cardiomyocytes expressed in the ventricles and brain tissue. BNP secreted from cardiomyocytes is present as a precursor of 108 amino acids. When cardiomyocytes are stimulated, BNP is cleaved into an inactive linear polypeptide comprising 76 amino acids and an active cyclic peptide consisting of 32 amino acids. Upon stimulation of an activating enzyme, the inactive linear polypeptide and the active cyclic peptide are released into the blood circulation, respectively known as NT-proBNP and BNP [5]. The active protein’s physiological effects include natriuresis, diuresis, vasorelaxation, inhibition of renin and aldosterone secretion, and contributions to cardiovascular homeostasis. High levels of BNP in the bloodstream suggest acute heart failure. According to data from the UK, a cutoff in BNP concentration of 100 pg/ml has a sensitivity of approximately 100%, a negative predictive value of approximately 100%, a specificity of 90%, and a positive predictive value of 78% for cardiac events [6].

In clinical practice, we observed that a certain number of patients presented with various degrees of edema and occasional chest pain after rhIL-11 injection. We suspect that this may be related to the effects of rhIL-11 on the circulatory system. Therefore, we designed this study to explore the correlation between rhIL-11 administration and BNP concentration in leukemia patients after receiving chemotherapy. The possible underlying mechanism of the effect of rhIL-11 on the circulatory system is also discussed.

## Methods

### Patients

Twenty-four adult patients diagnosed with leukemia who received chemotherapy between October 2016 and May 2017 at the Department of Hematologic Oncology, Cancer Center, Sun Yat-Sen University were enrolled. All patients had a confirmed diagnosis of leukemia according to the French-American-British (FAB) classification system based on clinical features, bone marrow cytology, flow cytometric evaluation, and cytogenetic detection. All patients had a Karnofsky performance status (KPS) score > 70. Patients with basic heart disease were excluded by ultrasound, electrocardiogram, and blood biochemistry.

### Treatment

Nine patients were diagnosed with acute lymphoblastic leukemia (ALL), seven of whom were treated with the MRC UKALLXII/ECOG E2993 protocol (from a joint international study initiated by the Medical Research Council in the UK and the Eastern Cooperative Oncology Group in the US in 1993) [22], and the other two received VDLP regimens. The remaining 15 patients with acute myeloid leukemia were treated with AML-201 [23].

### Dosing method

The patients underwent a bone marrow suppression period after chemotherapy. When their platelet count was ≤ 50 × 109/L, the patients were given a subcutaneous injection of rhIL-11 at 3 mg once a day for no more than 14 days until the platelet count was ≥ 20 × 109/L.

### Serum BNP measurement

We measured the serum BNP concentration 2–3 days before rhIL-11 treatment. During IL-11 administration, the patients’ BNP levels were re-examined twice a week. Finally, we measured the BNP concentration once again 2–5 days after withdrawal of rhIL-11 medication.

### Cell culture

H9C2 rat cardiomyocytes were cultured in 10% FBS at 37 °C with 5% CO_2_, and the medium was changed every 2–3 days.

### Real-time quantitative reverse transcription polymerase chain reaction

The BNP mRNA levels in cardiomyocytes were detected by real-time quantitative reverse transcription polymerase chain reaction (RT-PCR) according to the manufacturer’s instructions. The primer sequences used for RT-PCR were as follows: mouse BNP, forward 5′-TCCTAGCCAGTCTCCAGAGCAA-3′, reverse 5′-GGTCCTTCAAGAGCTGTCTCTG-3′; mouse GAPDH, forward 5′-CATCACTGCCACCCAGAAGACTG-3′, reverse 5′-ATGCCAGTGAGCTTCCCGTTCAG-3′; rat BNP:, forward 5′-AGCTGCTGGAGCTGATAAGAG-3′, reverse 5′-CGCCGATCCGGTCTATCTTC-3′; and rat GAPDH, forward 5′-CATCACTGCCACCCAGAAGACTG-3′, reverse 5′-ATGCCAGTGAGCTTCCCGTTCAG-3′. Real-time quantitative RT-PCR was performed for each sample in duplicate with GAPDH as a housekeeping gene. The comparative cycle threshold (Ct) method was used to determine the relative expression of BNP. Each sample was tested in triplicate.

### Statistical analysis

All statistical analyses were performed with SPSS software (version 13.0, SPSS Inc., Chicago, IL, USA). To make comparisons, the BNP index, blood pressure, and weight change before and after the use of rhIL-11 were estimated using a *t* test. The results of the comparative tests were considered significantly different if the two-sided *P* value was less than 0.05.

#### Data availability

Literature collection was performed through PubMed. All statistical analyses were performed using SPSS 19.0 or Graphpad Prism6.0. Data are stored in the corresponding author of this article and are available upon request.

## Results

### Patient characteristics

The clinical characteristics (including age and gender) of the 24 patients observed in this study are listed in Table 1. The ratio of males to females was 1.67:1, with a median age of 36.3 years (range, 19–66 years). All patients experienced grade IV myelosuppression after chemotherapy, and none of the patients had a history of heart disease.

**Table 1:** Patients characteristics

Patients no.	Age years	Gender	Diagnosis	Treatment	Initial serum BNP level	Mean serum BNP level during medication	Serum BNP level after medication	Clinical manifestation of HF	Initial weight	Weight during medication	Initial BP	BP during medication
1	22	Female	AML	AML201	0	109.94	38.11	No	31	32	128/86	135/89
2	34	Male	ALL	VDLP	21.08	79.28	26.23	No	58	57.5	128/71	134/80
3	23	Male	AML	AML201	0	35.91	10.5	No	65	65	120/71	128/75
4	29	Female	AML	AML201	5.35	131.10	41.64	No	47	47	105/65	110/70
5	36	Female	AML	AML201	0	207.76	12.04	No	48	48.5	103/75	110/78
6	49	Female	AML	AML201	20.33	63.81	7.74	No	57	56.5	113/74	121/82
7	22	Female	ALL	E2993	21.46	73.07	31.4	No	46	46.8	109/73	115/78
8	35	Female	AML	AML201	0	129.23	19.61	No	45.5	45	133/80	135/81
9	58	Male	AML	AML201	99.05	359.28	168.6	Yes	69	75	140/80	146/85
10	53	Female	CML	AML201	17.58	33.37	37.27	No	40	40	130/81	136/85
11	59	Female	AML	AML201	24.5	173.98	148.57	No	53	52	117/67	120/69
12	30	Female	ALL	VDLP	22.68	105.66	60.59	No	49	48.5	105/66	109/70
13	20	Male	ALL	E2993	4.93	44.29	35.89	No	74	74	118/72	124/75
14	19	Male	ALL	E2993	55.21	84.77	30.12	No	80	80	134/81	138/89
15	34	Female	AML	AML201	5.26	109.91	109.05	No	53	54	109/71	116/79
16	30	Male	ALL	E2993	8.72	19.08	9.56	No	71	70	122/75	128/78
17	32	Female	AML relapse	Cladribine+Aclarubicin	2.52	95.91	46.79	No	39	39	112/69	120/74
18	30	Male	ALL	E2993	27.4	35.68	33.33	No	75	74.5	135/79	138/84
19	53	Female	AML	Cladribine+Aclarubicin+Cytarabine	35.28	502.47	78.61	Yes	70	74	134/79	139/85
20	40	Female	AML	AML201	2	127.12	6.37	No	50	49.5	113/77	119/84
21	25	Female	AML	AML201	77.29	213.42	103.4	No	44	45	99/63	108/72
22	42	Male	AML	AML201	27.26	181.29	68.78	No	58	58	99/66	110/74
23	29	Female	ALL	E2993	4.11	899.71	84.36	Yes	55	57	128/82	136/88
24	66	Male	ALL	E2993	42.4	1343.97	466.14	Yes	64	67	133/79	137/88

### Variation in serum BNP before and after rhIL-11 administration

**Fig. 1:**
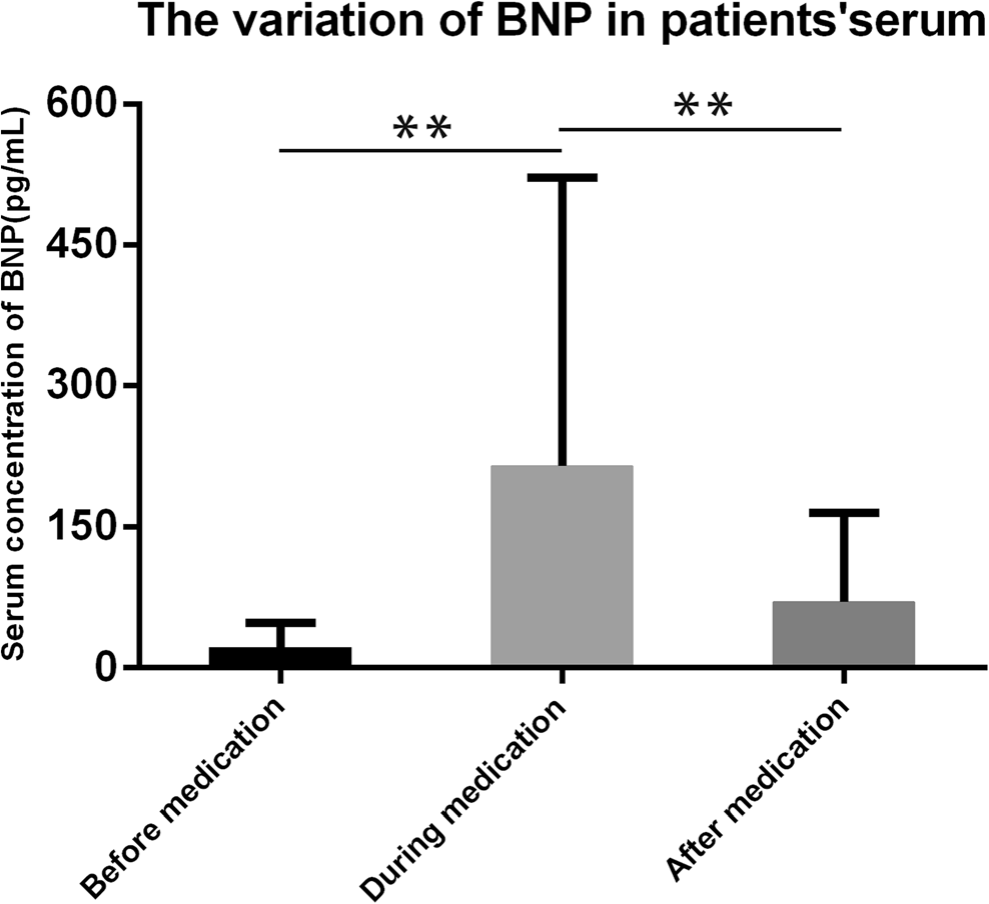
The mean concentration of serum BNP in leukemia patients was 215.00 pg/mL during rhIL-11 treatment, which was significantly higher than that before treatment (21.85 pg/mL). At 2–5 days after stopping rhIL-11 administration, the mean concentration of serum BNP returned to 69.78 pg/mL (mean ± SD; *P* < 0.001)

The mean concentration of serum BNP for all patients was 21.85 pg/mL (range, 0–99.05 pg/mL), with a median of 18.96 pg/mL before rhIL-11 treatment. During rhIL-11 treatment, the serum BNP of all the patients was surprisingly increased to an average concentration of 215.00 pg/mL, which was significantly higher than that before treatment (*P* < 0.001, Fig. 1). The BNP levels in 19 patients exceeded 100 pg/mL, which is regarded as the cutoff to predict heart failure. We tested BNP once again 2–5 days after stopping rhIL-11 treatment, and the mean serum BNP concentration returned to 69.78 pg/mL (*P* < 0.001, Fig. 1).

### Relationship between serum BNP levels and clinical features

**Fig. 2:**
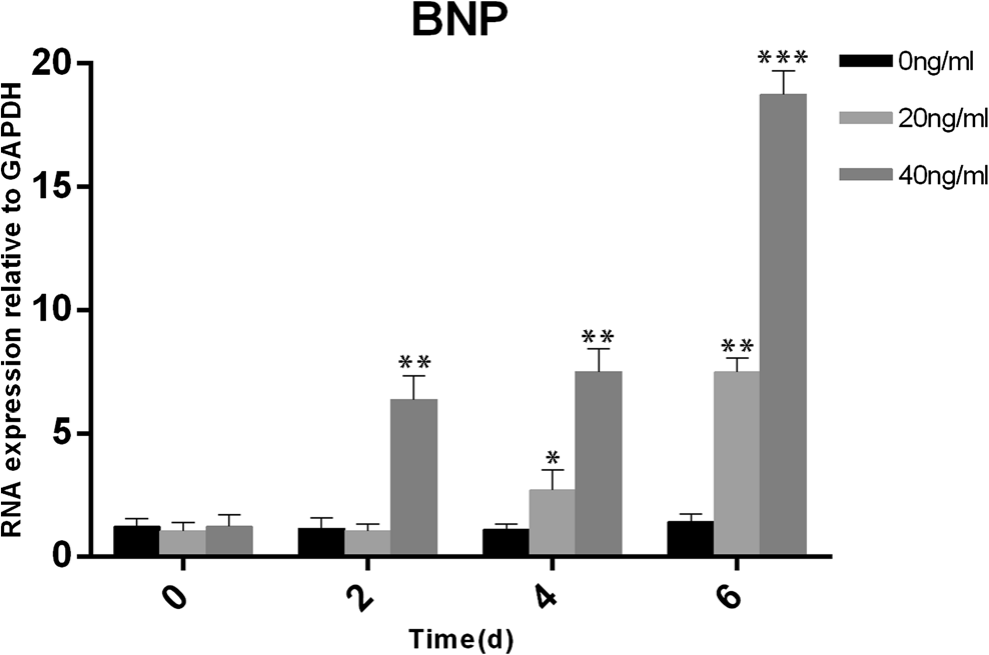
H9C2 cells were stimulated with rhIL-11 at a concentration of 0/20/40 ng/ml. BNP expression was determined by qRT-PCR at 48, 96, and 144 h after stimulation (mean ± SD). *P < 0.05, **P < 0.01, ***P < 0.001; Student’s *t* test

The concentration of serum BNP has been reported to increase when patients have sodium retention. The patients in this situation often presented leg edema and weight gain [7, 8]. Furthermore, edema is an important subclinical symptom of heart failure. In our study, nine patients had edema and weight gain, and four of them developed acute left heart failure. Additionally, two patients had paroxysmal atrial fibrillation. Heart failure in these patients was reversible and improved with careful medication regimens. It should be mentioned that there is an inseparable relationship between BNP and blood pressure [9–12]. The patients’ blood pressure was 119 ± 20 /75 ± 11 mmHg and 127 ± 19 /79 ± 10 mmHg before and after using rhIL-11, respectively (*P* < 0.005, Fig. 2).

### Change in BNP mRNA expression in the H9C2 cardiomyocyte cell line after rhIL-11 stimulation

We used different concentrations of rhIL-11 (0/20/40 ng/ml) to stimulate H9C2 cardiomyocytes and detected the mRNA expression of BNP with quantitative real-time RT-PCR. We found that the mRNA expression of BNP in cardiomyocytes was significantly increased, showing a positive correlation with the stimulation time and drug concentration (Fig. 2).

## Discussion

Several published studies have investigated the cardiotoxicity of rhIL-11, and most of the observed pathologies were reversible arrhythmia [13–16]. However, the concomitant elevation of BNP and incidence of heart failure are seldom mentioned. Based on our clinical observations and monitoring, it was no coincidence between elevated BNP levels and increases in their associated clinical symptoms caused by rhIL-11. During rhIL-11 administration, the serum BNP and blood pressure of all the patients were increased. Nine patients had edema and weight gain, and four of them presented acute left heart failure.

To further validate our results, we conducted a series of in vitro cell-based experiments. In cardiomyocytes stimulate with rhIL-11, the mRNA levels of BNP were increased, which is consistent with our clinical observations. Studies have shown that cytokines of the IL-6 family induce cardiomyocyte hypertrophy [17–20] resulting in increased ANP and BNP expression and activation of the p42/p44-MAPK and PI3K signaling pathways may be related [21]. Since IL-11 is a member of the IL-6 family, we infer that IL-11 can also cause elevated BNP via the cardiac hypertrophy pathway.

The use of adjuvant therapy is critical for leukemia patients undergoing chemotherapy. rhIL-11 is widely used to treat grade III or grade IV thrombocytopenia (i.e., platelets ≤ 50 × 109/L). As a result, the number of thrombocytes is indeed increased, and the risk of bleeding is reduced. However, the side effects of rhIL-11 cannot be ignored. Through our clinical observations and experiments, we conclude that the use of rhIL-11 can either directly or indirectly induce cardiotoxicity. BNP is a good marker for cardiotoxicity, and we recommend routine detection of BNP when administering rhIL-11. Daily measurement of blood pressure and body weight is also suggested. We do not advise the use of rhIL-11 in any patients with conditions that may cause or exacerbate existing heart failure, such as underlying heart disease, pulmonary infection, or other situations. Caution should be taken when considering rhIL-11 use in older and more frail patients.

The main limitation of our experiment is that the sample size of this study is small, and our task in the next stage is to expand the cohort to further confirm the results of this study. Additionally, more cell experiments can verify our conclusions. For example, secreted BNP protein levels can be detected by ELISA, and pathway proteins can be detected by Western blot. We will expound our observations in the follow-up study and explore the mechanism of rhIL-11-induced cardiotoxicity in greater depth.
